# Supplementary material for: Performance of Washing-Free Printing of Disperse Dye Inks: Influence of Water-Borne Polymers
Source: Polymers (Basel). 2022 Oct 12;14(20):4277. doi: 10.3390/polym14204277 (PMC9610862; doi:10.3390/polym14204277)
Supplement: Supplementary file 1 [file polymers-14-04277-s001.zip › polymers-1912070-supplementary.pdf]

Supplementary Materials

Disperse yellow dye inks were prepared by the methods of 2.2. and 2.3., named Y-PVP, Y-PVA, Y-PEG, Y-Control, respectively. C.I. Disperse Yellow 48 (press cake) was obtained from Annoqi Group Co., Ltd. (Shanghai, China). Table S1. shows the physicochemical properties of disperse yellow dye inks. To avoid clogging of the print head, the particle size of disperse yellow dye ink is kept below 200 nm, and the conductivity is far lower than the 104 μS/cm required by the ink. The zeta potential, surface tension and pH value of the four inks fell within the normal range for commercial inks.

Table S1. Physicochemical properties of disperse yellow dye inks.

Sample	Particle size (nm)	Zeta potential (mV)	pH	Conductivity (μS/cm)	Surface tension (mN/m)	Viscosity (mPa·s)
Y-PVP	175.3	−44.5	8.03	6.21	32.37	1.95
Y-PVA	157.6	−51.2	7.98	6.13	33.08	2.06
Y-PEG	161.6	−43.4	8.04	6.15	32.28	2.08
Y-Control	157.9	−45.6	7.87	6.03	32.93	1.94

On the basis of 3.3., the correlation between shear rate and viscosity of disperse blue dye inks at different temperatures was tested. As shown in Table S2., the intermolecular force weakens with the increase of temperature, and the ink fluidity increases and viscosity decreases. And the viscosity are summarized in Table S2.

Table S2. The viscosity of the disperse yellow dye inks at different temperatures.

Sample	20 °C (mPa·s)	25 °C (mPa·s)	30 °C (mPa·s)	35 °C (mPa·s)
PVP	2.31	1.98	1.76	1.58
PVA	2.31	1.90	1.70	1.53
PEG	2.56	2.28	2.00	1.79
Control	2.06	1.77	1.57	1.40

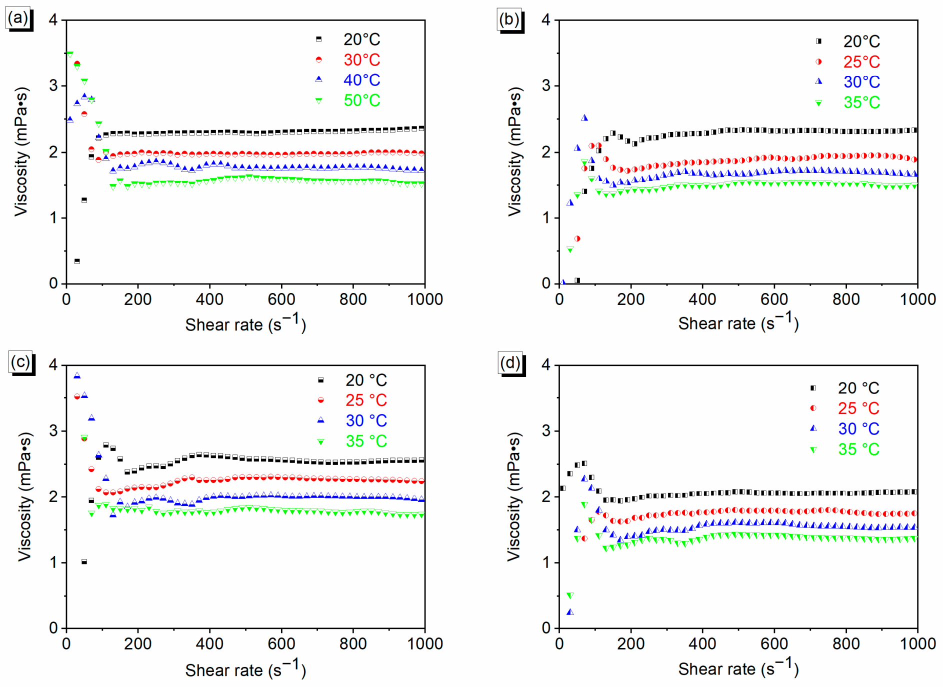

Figure S1. Correlation between shear rate and viscosity of disperse yellow dye inks at different temperatures: (**a**) PVP ink; (**b**) PVA ink; (**c**) PEG ink and (**d**) Control sample.

Figure S2 (a) shows the viscosity curves of ink solutions at different shear rates. The viscosities of the inks were 1.95, 2.06, 2.8 and 1.94 mPa·s, respectively. Figure S2 (b) shows the shear stress of the ink as a function of shear rate. It can be seen that the inks were all typical Newtonian fluids.

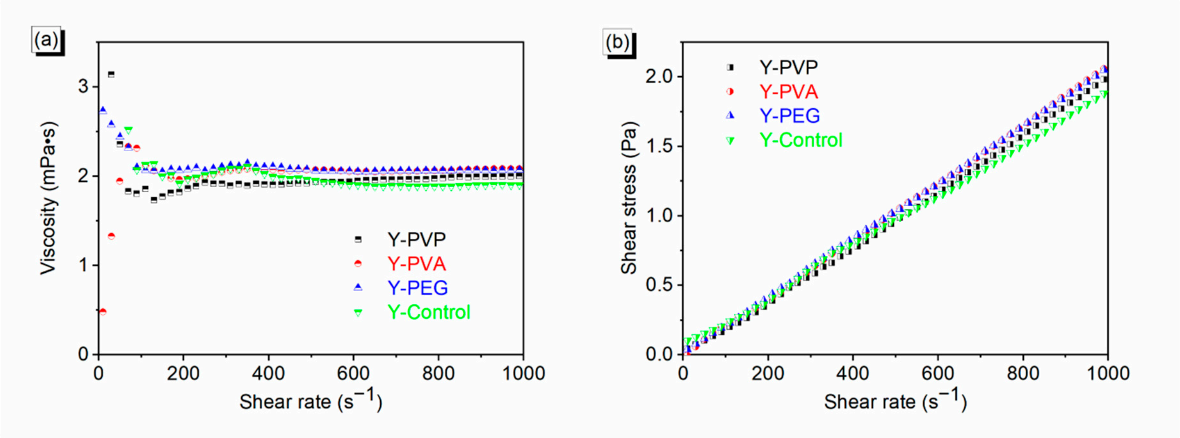

Figure S2. (**a**) Correlation between shear rate and viscosity of disperse yellow dye inks with different additives and (**b**) correlation between shear rate and shear stress.

As shown in Figure S3., the disperse yellow dyes exhibited different degrees of diffusion on the PET films during thermofixation. Relative to that of the Y-control sample, the migration of the ink with added water-borne polymer in the PET film was enhanced, and the effect was the most obvious for the Y-PVA ink.

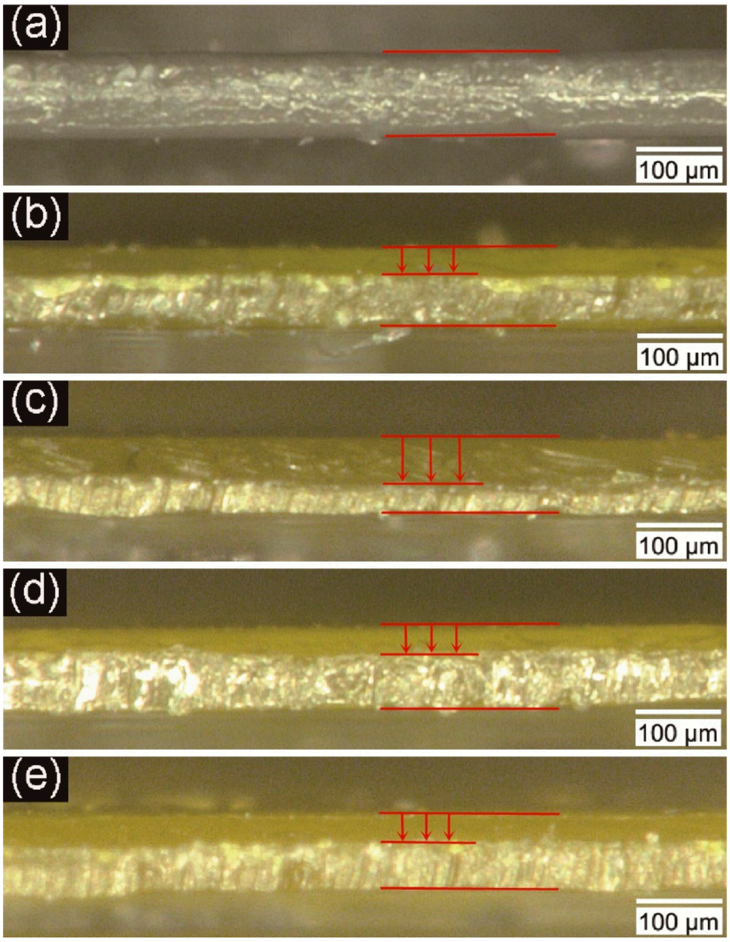

Figure S3. Cross-section images of printed PET films: (a) pure PET film; (b) PET film printed with Y-PVP ink; (c) PET film printed with Y-PVA ink; (d) PET film printed with Y-PEG ink; and (e) PET film printed with Y-Control sample.

Figure S4. shows an SEM images of pristine polyester fabric and polyester fabric printed with Y-PVP ink, Y-PVA ink, Y-PEG ink and Y-Control sample. Because PVA has good film-forming performance at room temperature, the surface of fabrics printed with Y-PVA ink is more consistent and smoother.

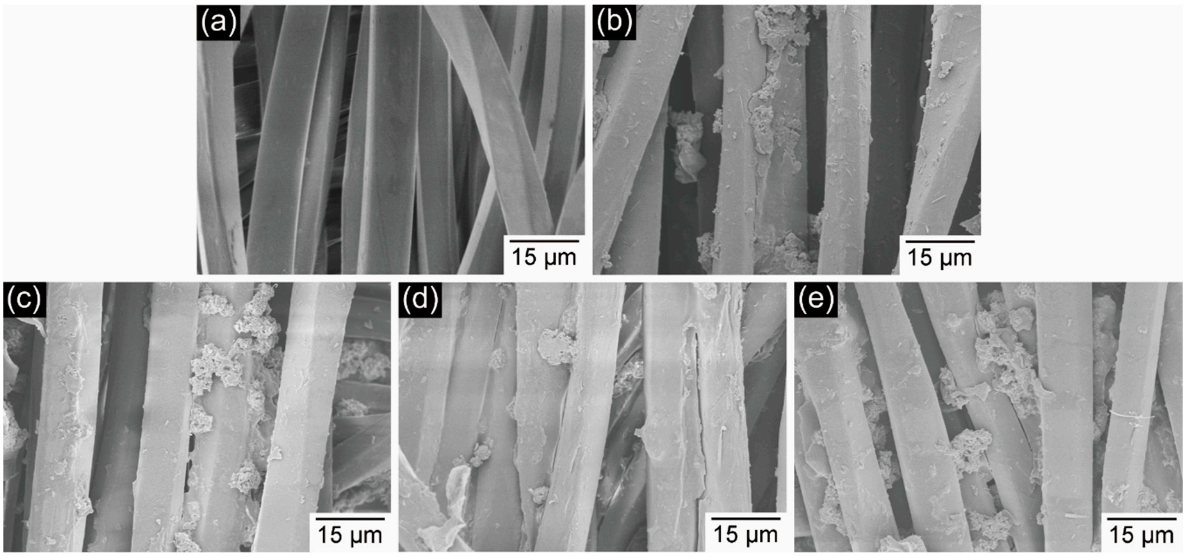

Figure S4. SEM images of fabrics: (**a**) polyester fabric; (**b**) polyester fabric printed with Y-Control sample; (**c**) polyester fabric printed with Y-PVP ink; (**d**) polyester fabric printed with Y-PVA ink; and (**e**) polyester fabric printed with Y-PEG ink.

Table S3. shows the color strength and color fastness data of the printed samples. The K/S values of the samples are 5.03 (Y-PVP ink), 5.53 (Y-PVA ink), 4.13 (Y-PEG ink), and 4.30 (Y-Control sample). The rubbing fastness of the printed samples reached grade 3–4, and that of Y-PVA sample reached grade 4. The washing fastness of the samples reached grade 4–5.

Table S3. Color yield and color fastness of the printed polyester fabric using disperse yellow dye inks with different additives and control sample.

Sample	K/S	Rubbing Fastness		Washing Fastness
		Dry	Wet	
Y-PVP	5.03	3–4	3–4	4–5
Y-PVA	5.53	4	4	4–5
Y-PEG	4.13	3–4	3–4	4–5
Y-Control	4.30	3–4	3–4	4–5

Table S4. shows the line width and linewidth relative change (W) of the printed samples. The water-borne polymer-added samples demonstrated better print clarity than the control sample. Among the inks, the PVA ink exhibits excellent printing clarity.

Table S4. Linewidth and linewidth relative change (W) of printed fabric.

Sample	Printing Pattern	Warp Linewidth (μm)	W (%)	Printing Pattern	Weft Linewidth (μm)	W (%)
Y-PVP	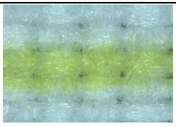	441.25	25.00	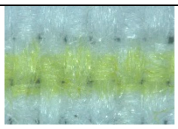	392.06	11.07
Y-PVA	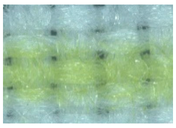	456.93	29.44	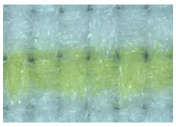	382.00	8.22
Y-PEG	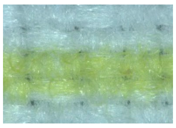	440.15	24.69	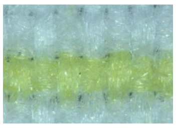	414.66	17.47
Y-Control	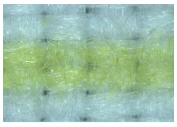	468.23	32.64	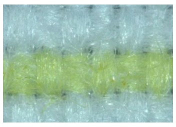	416.35	17.95
